# 24-hour movement behaviours and the risk of common mental health symptoms: A compositional analysis in the UK biobank

**DOI:** 10.1192/j.eurpsy.2021.341

**Published:** 2021-08-13

**Authors:** A. Kandola, B. Del Pozo Cruz, D. Osborn, B. Stubbs, K. Choi, J. Hayes

**Affiliations:** 1 Division Of Psychiatry, University College London, London, United Kingdom; 2 Institute For Positive Psychology And Education, Australian Catholic University, Sydney, Australia; 3 Institute Of Psychiatry, Psychology, And Neuroscience, King’s College London, London, United Kingdom; 4 Department Of Psychiatry, Massachusetts General Hospital, Boston, United States of America

**Keywords:** sedentary behaviour, Physical Activity, Depression, Anxiety

## Abstract

**Introduction:**

Sedentary behaviour is potentially a modifiable risk factor for depression and anxiety disorders, but findings have been inconsistent.

**Objectives:**

To assess associations of sedentary behavior with depression and anxiety symptoms and estimate the impact of replacing daily time spent in sedentary behaviors with sleep, light, or moderate-to-vigorous physical activity, using novel compositional data analysis methods.

**Methods:**

Prospective cohort study in with 60,235 UK Biobank participants (mean age: 56; 56% female). Exposure was baseline daily movement behaviours (accelerometer-assessed sedentary behaviour, physical activity, and self-reported total sleep). Outcomes were depression and anxiety symptoms (Patient Health Questionnaire-9 and Generalised Anxiety Disorders-7) at follow up.

**Results:**

Replacing 60 minutes of sedentary behaviour with light activity, moderate-to-vigorous activity, and sleep was associated with lower depression symptom scores by 1·3% (95%CI, 0·4%-2·1%), 12·5% (95%CI, 11·4%-13·5%), and 7·6% (95%CI, 6·9%-8·4%), and lower odds of depression by 0·95 (95%CI, 0·94-0·96), 0·75 (95%CI, 0·74-0·76), and 0·90 (95%CI, 0·90-0·91) at follow-up. Replacing 60 minutes of sedentary behaviour with moderate-to-vigorous activity and sleep was associated with lower anxiety symptom scores by 6·6% (95%CI, 5·5%-7·6%) and 4·5% (95%CI, 3·7%-5·2%), and lower odds of meeting the threshold for an anxiety disorder by 0·90 (95%CI, 0·89-0·90) and 0·97 (95%CI, 0·96-0·97) at follow-up. However, replacing 60 minutes of sedentary behaviour with light activity was associated with higher anxiety symptom scores by 4·5% (95%CI, 3·7%-5·3%) and higher odds of an anxiety disorder by 1·07 (95%CI, 1·06-1·08).

**Conclusions:**

Sedentary behaviour is a risk factor for increased depression and anxiety symptoms in adults, but different replacement activities differentially influence mental health.

**Disclosure:**

No significant relationships.

